# Occurrence of microplastic, antibiotics, hormones, and heavy metals in livestock and poultry manure in west iran

**DOI:** 10.1038/s41598-025-03826-7

**Published:** 2025-07-26

**Authors:** Mitra Mohammadi, Abdollah Dargahi, Ali Almasi, Seyyed Alireza Mousavi, Parviz Mohammadi

**Affiliations:** 1 Student Research Committee, Kermanshah University of Medical Sciences, Kermanshah, Iran; 2 Social Development and Health Promotion Research Center, Health Policy and Promotion Institute, kermanshah, Iran; 3 Department of Environmental Health Engineering, Khalkhal University of Medical Sciences, Khalkhal, Iran; 4 Social Determinants of Health Research Center, Ardabil University of Medical Sciences, Ardabil, Iran; 5 Department of Environmental Health Engineering, School of Public Health, Kermanshah University of Medical Sciences, Kermanshah, Iran

**Keywords:** Microplastic, Emergent pollutants, Livestock manure, Poultry manure, Biological techniques, Systems biology, Environmental sciences

## Abstract

Identifying emerging contaminants in animal manure has great importance in sustainable development. Therefore, in this study, microplastics, antibiotics, hormones, and heavy metals in cow (COM) and poultry manure (POM) were investigated in Kermanshah province, which is one of the hubs of livestock and poultry farming. A total of 71.42% of the samples contained microplastics with four morphotypes: fiber, film, fragment, and sphere. These microplastics were characterized by colorless, green, yellow, blue, white, and black hues. PE and PP were the maximum composition. The abundance and diversity of microplastics in COM were higher than in POM. The concentration of doxycycline, erythromycin, enrofloxacin, and progesterone was in the range of 0.53–2.3 mg/kg, 0.49–1.9 mg/kg, 0.54–2.6 mg/kg, and 46.2–21.5 mg/kg, respectively. Zn (mg/kg) in COM and POM was 248.16 ± 31.62 and 200.1 ± 39.17, respectively, which were the maximum heavy metals. The average of Cr and Cu in COM was recorded as 34.46 ± 17.58 and 100.2 ± 2.22.03, and in POM as 34.2 ± 11.6 and 148.4 ± 57.02, respectively. The direct application of COM and POM could be a new route for the presence of emerging pollutants in agricultural soils. Therefore, this issue should be given serious attention by the environmental officials in Iran, and a solution should be devised.

## Introduction

There is a close interrelationship between health and sustainable development^1^. In recent years, increasing demand for organic products has led to the application of animal manure (AMs) instead of chemical fertilizers^2^. Iran’s agricultural land under organic cultivation amounts to 31–33 million hectares, mostly managed traditionally by smallholder farmers. Since 1990, AMs production has doubled in Africa, while Asia and the Americas have seen the highest levels of AMs production^3^. 57 million tons of livestock manure are produced in Iran, annually, while over 744773.760 tons are produced for poultry farms^4^. According to the Li et al.(2020) study^5^, this level is 47 million tons in Korea, 1.4 billion tons in the European Union, and 3.8 billion tons in China. Using AMs in agricultural lands as organic fertilizer, despite its advantages, may also be a source of Environmental Pollutants (EPs) that have adverse effects on human health and the environment^6^. Important pollutants in AMs are heavy metals, antibiotics^7^, hormones^8^, and plastics^9^. The presence of pharmaceutical compounds in animal manures has been investigated in various countries^10–13^.In Beriot et al. (2021)^14^ in Spain, the potential transport of microplastics by a flock of 1,000 sheep was estimated to be about 106 particles per hectare per year. Studies in China^15,16^ Italy^17^, and Tunisia^10^, confirmed the presence of MPs in AMs. On the other hand, HMs studies in recent years have shown the presence of chromium^18^, lead^19^, zinc^20^, cadmium^21^, arsenic^22^, etc., in animal manure. Unfortunately, in Iran, the presence of EPs in various sources, including AMs, has not received sufficient attention. The lack of attention in policymaking for this category of pollutants will lead to health, environmental, and even economic impacts for the country^23^. Studies on EPs and their impact on human health and the environment have received more attention in recent years^24,25^. Soil pollution by livestock waste can, in addition to reducing soil fertility and contaminating water resources, enter the food chain of humans and other organisms by being absorbed by plants^26^.

Among the challenges in the use of AMs in agricultural soils is the release of pharmaceutical compounds such as antibiotics and hormones into the environment. Most of these pharmaceutical compounds are poorly absorbed in the digestive system of animals, so that about 25–75% of them are ultimately excreted undigested^27,28^. The major part of the release of these pharmaceutical compounds is due to the application of fresh or composted AMs in agricultural lands, which in the long term leads to increased resistance of pathogens. This is mainly done by creating antibiotic resistance genes^29^. Available information indicates the adverse effects of antibiotics on the soil ecosystem and their effect on soil microorganisms. Soil microorganisms and plants are closely related to each other, therefore, any destructive effect that destroys the balance between microorganisms will lead to adverse effects on soil fertility and sustainability of the ecosystem^30^.

Microplastics (MPs) are a global environmental concern due to their widespread contamination, which may pose risks to food security and human health after entering agricultural soils^31^. Microplastics can alter the physicochemical properties of soil, such as its structure, water holding capacity, and density, which can limit root growth, nutrient uptake, and crop yield^32^. Soil microplastics can be transferred to humans through the food chain or water cycle, posing long-term risks to human health^33^.

Heavy metals (HMs) and metalloid pollution have become a serious problem in agriculture^34^. HMs accumulate in agricultural soils without obvious toxic effects, can be released into water, absorbed by crops, and enter the food chain in high concentrations, causing negative effects on human and animal health. The main input of heavy metals is animal feed, which must be controlled to prevent excessive spread of HMs in the environment^35^.

Despite the widespread use of AMs in agricultural soils in Iran, information on the presence of microplastics, hormones, antibiotics, and heavy metals in AMs in Iran is limited. Consequently, the current investigation, which is designed to investigate the presence of microplastics, hormones, antibiotics, and heavy metals in cow and poultry manure, is of the utmost importance and is essential for the implementation of appropriate policies and the effective management of these pollutants in Iran.

## Materials and methods

### Site description and sample collection

Kermanshah province in western Iran, with an area of ​​24,640 km^2^, is one of the centers of livestock and poultry production in Iran, with more than 1,333 industrial livestock and 57 poultry farming units. In addition to industrial units, a large number of livestock and poultry are raised by villagers and nomadic tribes in Kermanshah province and exported to other provinces. Sampling was carried out from September 2023 to March 2024. A total of 19 samples of 50 g were taken and immediately transferred to the laboratory. The sampling method was in a zigzag pattern (Z), so that 15 to 20 samples were taken from each location with the help of a shovel in a zigzag pattern and then combined to obtain a combined sample. Then the sample was placed in a sealed glass container. The samples were obtained from 3 dairy farms (A, B, C) and 3 poultry farms (D, E, F) in Kermanshah province. Four samples were taken from site A, and 3 samples from other sites, with three repetitions. The geographical characteristics of the dairy farms are as follows: A:47.0548,34.3850, B:46.7643,34.2594, C:47.5377,34.7284. The geographical characteristics of the poultry farms are as follows: D:46.35.39,34.8813, E:46.8699,33.8973, and F:47.3242,34.3705. Animal antibiotic (AABs) and progesterone (PST) samples were stored at −20 °C, heavy metals (HMs) at 4 °C, and plastic at ambient temperature until the experiments were performed.

### Chemicals, sample preparation, and analysis

All chemicals and reagents used were analytical reagent grade (Merck, Germany). Reference standards for antibiotics of doxycycline hyclate ≥ 99%, erythromycin ≥ 850 µg/mg potency, enrofloxacin 99%, and progesterone 99.1% were purchased from Merck (Germany). The dry weight of the samples (Three composites) was determined according to the (Khare et al., 2023) method^36^. To prevent contamination, all tools were washed with double-distilled water and dried.

Study Ho et al.(2012)^37^ was used to determine whether the antibiotics doxycycline (DXC), erythromycin (ETC), and enrofloxacin (EFC), as well as progesterone (PST). For the determination of heavy metals (HMs) using wet digestion techniques, 0.5 g of the sample was combined with 3.5 mL of concentrated H_2_SO_4_. The mixture was left at room temperature for 30 min. H_2_O_2_ 30% (3.5 mL) was added and exposed to heat at 250 °C for 30 min. 1 mL of 30% H_2_O_2_ was added until it became clear. It was then filtered via Whatman filter paper No. 42. The copper (Cu), chromium (Cr), and zinc (Zn) were determined by atomic absorption spectrometry^38^. Extraction was performed using the method Wu et al.(2021)^39^. A centrifuge tube was used to digest the organic matter in the sample (3 g). Subsequently, 10 mL of 30% H_2_O_2_ and 1 mL of 10% FeSO_4_ solution were introduced to neutralize any excess H_2_O_2_. During digestion, samples were placed in cold water. In the next step, the samples were heated in a water bath at 50 °C for 2 h, and then 1 mL of 10% FeSO4 was added. To each sample, NaOH (0.5 M, 30 mL) was added, and after 24 h, it was sonicated. Saturated NaI solution (20 mL) was added and centrifuged for 10 min at 8500 rpm. A Whatman filter was used to filter the supernatant. The filter was then cleaned with 100% ethyl alcohol and allowed to dry on glass slides at room temperature. Morphology and composition of MPs were determined by a stereomicroscope and an FTIR spectrometer, respectively.

### Statistical analysis

The measured parameters were analyzed using SPSS software version 28 (SPSS, Inc., Chicago, IL). The data normality test is assessed by the Kolmogorov-Smirnov test. The Kruskal-Wallis and ANOVA test was used for nonparametric and parametric data, respectively, at α < 0.05.

## Results

### Occurrence of MPs in AMs

**Fig. 1:**
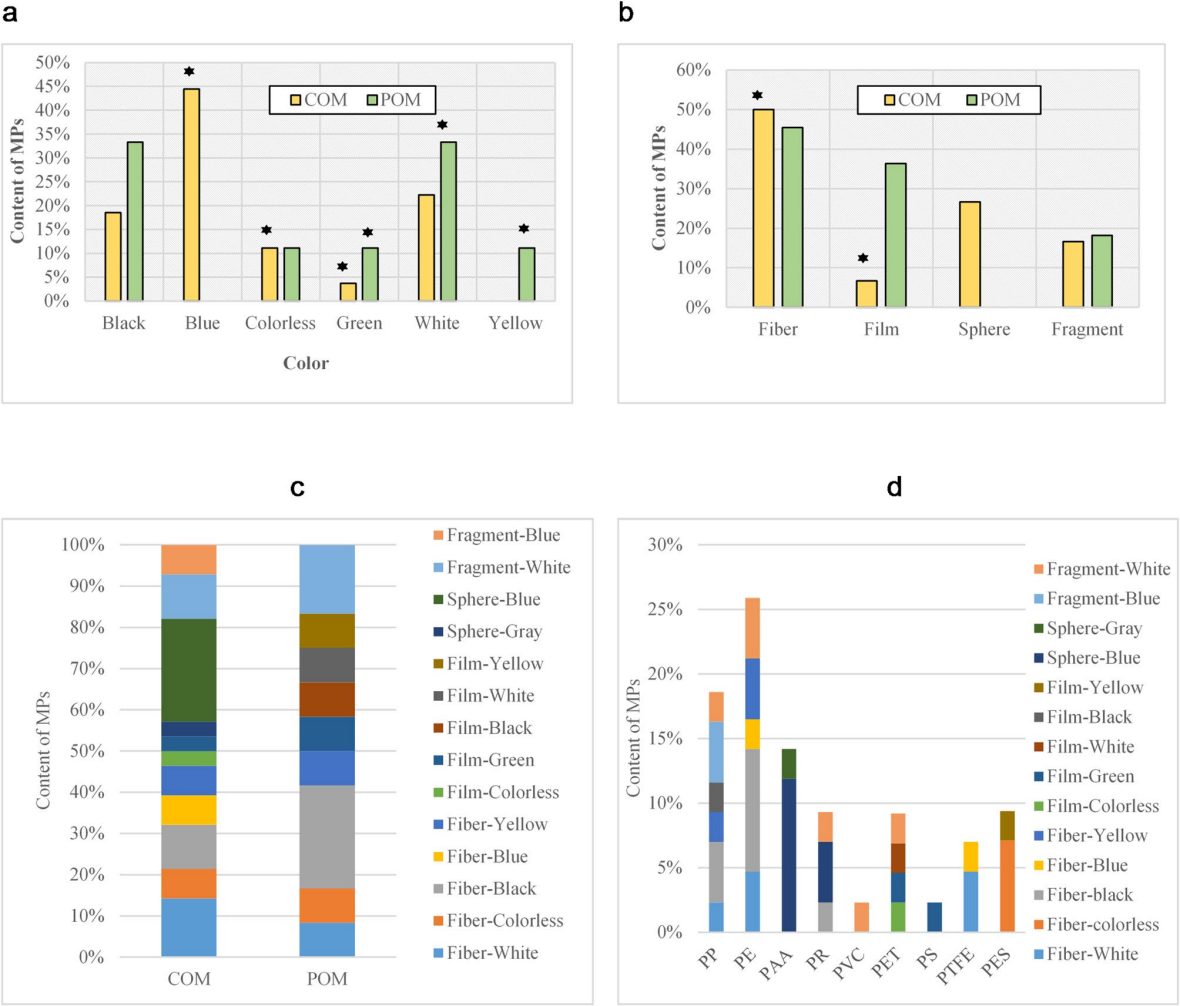
Distribution of MPs in COM and POM. **a**: color, **b**: shape, **c**: morphotype, and **d**: polymer type number in AMs.

MPs were found in 71.42% of samples. The distribution of MPs did not follow a normal distribution (Pvalue < 0.05). Concerning morphotypes, MPs showed a variety of shapes and colors. A total of 41 particles containing 4 shapes and 6 colors were identified in all samples (Fig. 1a, b). Fibers were reported in 5 colors, while fragments and film were seen in 2 and 5 colors, respectively. Sphere was reported in blue and gray. Blue spheres were the most abundant MPs in different manures. Regarding the proportion of different shapes and colors, the most morphotypes in COM were fiber shape (50%) and blue color (44.44%); and in POM, fiber (45.45%) and black and white (33.33% each) were maximum. Compared to the total number of MPs, the MPs contamination level in POM was lower than COM (Pvalue < 0.05). Sphere- blue (25%) and fragment-white (16.66%) were the most abundant morphotypes in COM and POM (Fig. 1c). There was no contamination in samples 8, 9, 12, 14, 17, and 21, while in most sites, only one MP was detected. Sample 7 contained the greatest number of items (5 items), indicating a statistically significant difference from the other samples (Pvalue < 0.05). The composition of the MPs indicated that polypropylene (PP) and polyethylene (PE) had the highest composition (21.95% each), while PVC had the lowest (2.43%). In general, compounds identified included PP, PE, PAA, PR, PVC, PET, PS, PTFE, and PES (Fig. 1d). PP in the morphotypes of 1 white fiber, 6 PAA were observed only in sites A and C as blue balls. As observed for MP polymer composition, there was a significant difference between the PP, PE, and PAA and other species. In poultry manure, PES, PTFE, PS, PET, PE, and PP were detected, while in cow manure, more compositions were present in different proportions. There was a significant difference between PP, PE, and PAA with others (Pvalue < 0.05). In poultry manure, PES, PTFE, PS, PET, PE, and PP were identified, while in COM, more compositions were present in different proportions.

### Occurrence of PST and AABs in AMs

**Fig. 2:**
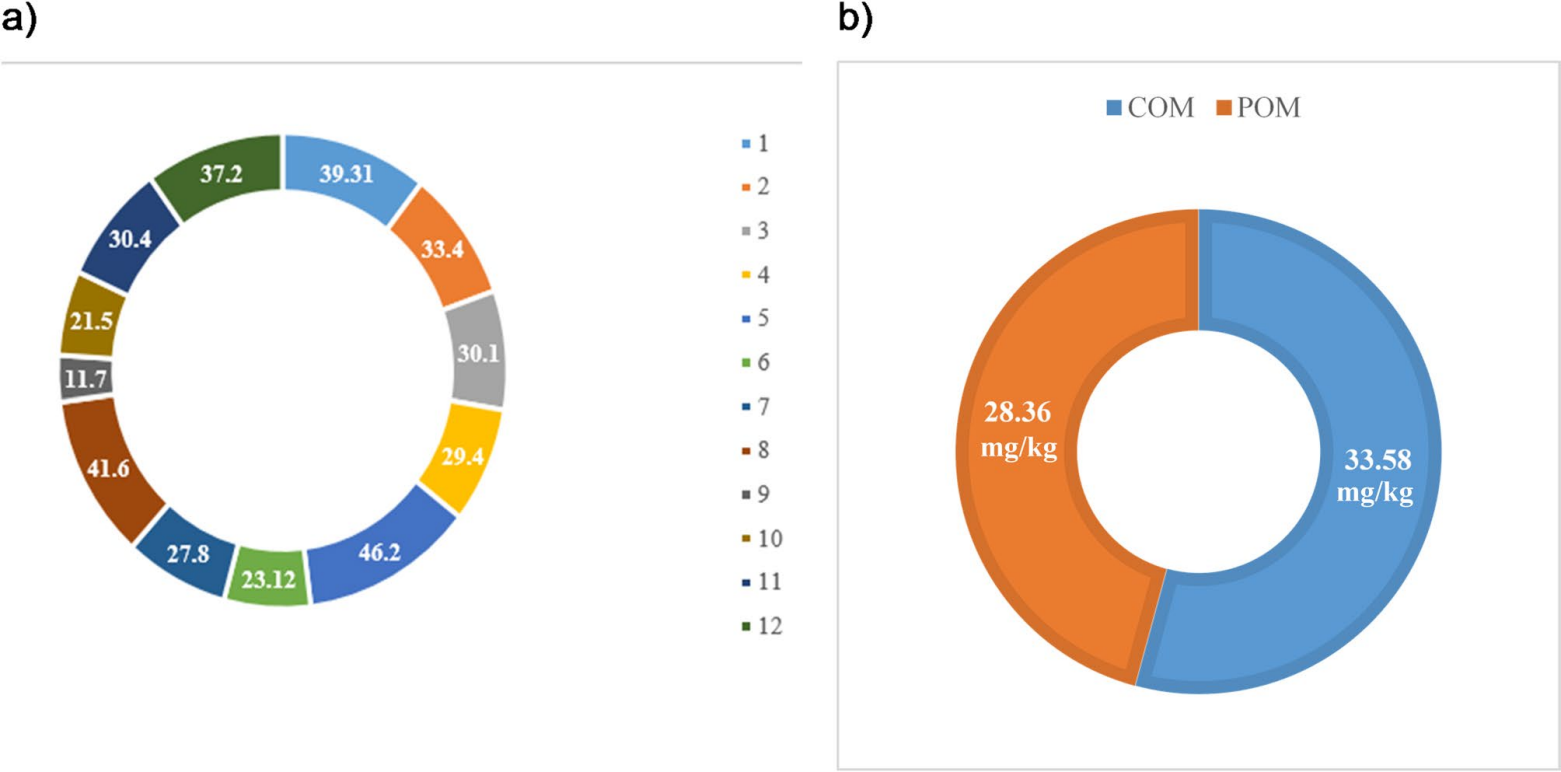
Progesterone concentration (mg/kg) in **a**) different samples and **b**) COM and POM.

As shown in Fig. 2, the presence of PST was confirmed in all samples (Table 1). However, the concentrations of PST varied among animal species (Pvalue < 0.05). The average PST concentrations at different sites in COM and POM were 33.58 ± 8.13 mg/kg and 28.36 ± 10.7 mg/kg, respectively. The maximum and minimum PST concentrations were in sites C (46.2 mg/kg) and E (11.7 mg/kg), respectively (Pvalue < 0.05).

**Table 1:** Progesterone content in present study.

Manure	Mean	S.D	Maximum	Minimum
POM	28.36*	10.79	37.2	11.7
COM	33.58*	8.13	46.2	29.4

*The difference is significant (Pvalue < 0.05).

**Fig. 3:**
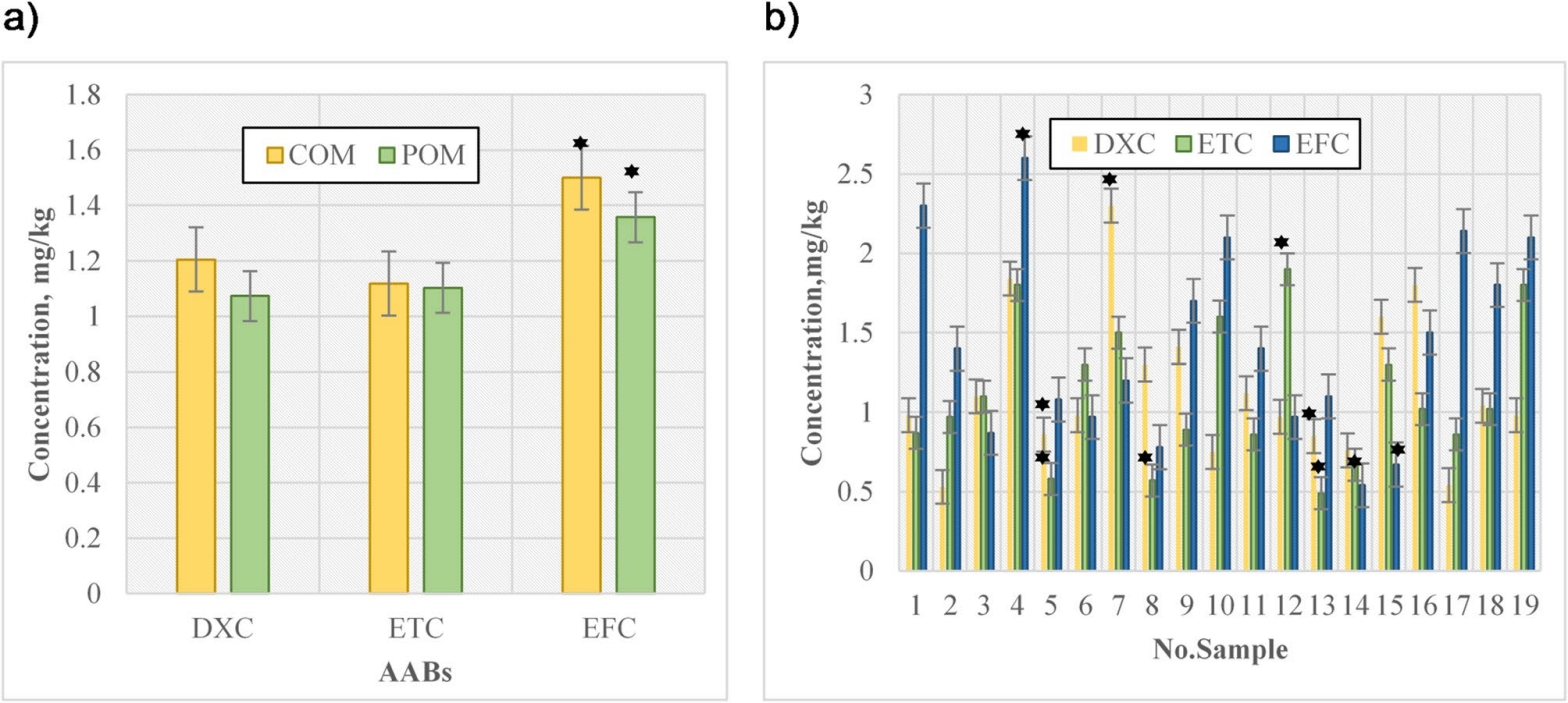
AABs in **a**) COM and POM and **b**) different sites in Kermanshah province.

AABs concentrations greatly varied across regions and animal species (Fig. 3b; Table 2). Based on dry weight, the average concentration (mg/kg) of DXC, ETC, and EFC in different sites for COM were 1.2 ± 0.53, 1.11 ± 0.42, and 1.5 ± 0.64, respectively, and for POM were 1.07 ± 0.39, 1.1 ± 0.47, and 1.32 ± 0.63, respectively. The maximum concentrations of DXC, EFC, and ETC were observed in sites E (1.38 mg/kg), F (2.01 mg/kg), and F (1.22 mg/kg), respectively. No significant differences were observed between the concentrations of ETC and EFC (Pvalue > 0.05), although the difference between DXC in POM and COM was significant (Pvalue < 0.05). Additionally, a statistically significant difference between the locations was noted. The minimum concentration (mg/kg) of DXC was at site F (0.85 ± 0.27 mg/kg); ETC and EFC were at site E (0.99 ± 0.31 and 0.9 ± 0.82), respectively. The concentration range of DXC (mg/kg) was reported to be 0.85–1.38, ETC 0.99–1.22, and EFC 0.9–2.01.

**Table 2:** Animal antibiotics content in the present study.

Manure	DXC, mg/kg				ETC, mg/kg				EFC, mg/kg			
	Mean	S.D	Maximum	Minimum	Mean	S.D	Maximum	Minimum	Mean	S.D	Maximum	Minimum
POM	1.07*	0.39	1.82	0.47	1.10	0.48	1.90	0.49	1.27	0.58	2.14	0.54
COM	1.2*	0.53	2.31	0.53	1.11	0.42	1.80	0.58	1.51	0.64	2.62	0.78

*The difference is significant (Pvalue < 0.05).

### Occurrence of HMs in AMs

**Fig. 4:**
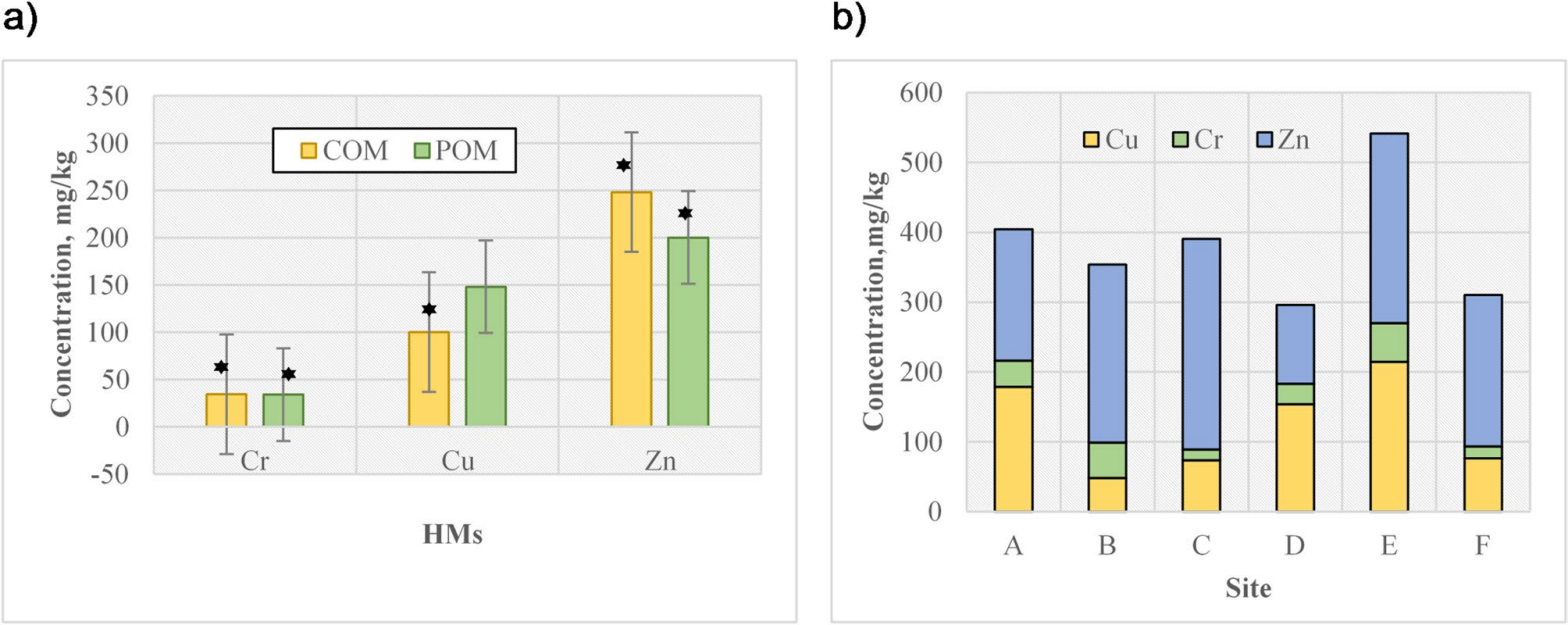
Heavy metal content in **a**) COM and POM and **b**) different sites in Kermanshah province.

HMs were recorded in high concentrations in all samples (Fig. 4; Table 3). Zinc (Zn) (248.16 mg/kg) in COM and Cu (148.1 mg/kg) in POM had the maximum concentrations. Cr concentrations in POM and COM were not significantly (Pvalue > 0.05), but Zn and Cu were significant (Pvalue < 0.05). HMs concentrations were the following trend: Zn > Cu > Cr. The maximum concentrations of Cu (214.3 mg/kg) and Cr (55.7 mg/kg) were observed in site E, and Zn (301.7 mg/kg) in site C. The minimum concentrations (mg/kg) of Cr (17.3), Cu (76.3), and Zn (271.2) were observed in POM in sites F, F, and E, respectively, and in COM in sites C, B, and A, respectively.

**Table 3:** Heavy metals content in the present study.

Manure	Zn, mg/kg				Cu, mg/kg				Cr, mg/kg			
	Mean	S.D	Maximum	Minimum	Mean	S.D	Maximum	Minimum	Mean	S.D	Maximum	Minimum
POM	200.1*	80.62	214.3	76.3	148.1*	69.17	214.3	48.6	34.2	19.6	50.4	15.6
COM	248.16*	57.02	301.7	112.5	100.2*	69.03	178.6	48.5	34.46	17.58	50.4	15.6

*The difference is significant (Pvalue < 0.05).

## Discussion

In the current study, emergent pollutants, especially MPs, in different animal manure were assessed. Results indicating that MPs, AABs, PST and HMs contamination of animal manure could no longer be neglected. To date, no study has been found that has surveyed MPs and HRs in cow and poultry manure in Iran, and the results were remarkable.

The presence of EPs such as MPs is currently a potential threat to the use of animal manure. Most importantly, in the future, as the MPs and other contaminants increase, more attention should be paid to the safety of animal fertilizer. The survey of microplastics in animal manure, PP, and PE was the most abundant composition. This was consistent with the report Geyer et al.(2017)^40^, which showed PP and PE are the most commonly used plastic compounds in the world. Furthermore, in the study Wu et al.(2021)^39^, the presence of MPs in AMs similar to the present study, PP, and PE were the most identified compositions. This finding suggests that MP contamination in animal husbandry environments cannot be disregarded, and that this contamination is present in AMs used as fertilizer in agriculture. Therefore, our results showed that applying COM and POM directly could be a route for MPs’ entrance into agriculture and compost production.

The source of PR may be from plastic adhesives for repairing and building water storage tanks, and unfortunately may contain bisphenol A. Bisphenol A was shown to affect reproductive traits, endocrine disruption^41^, the dysregulation of hypothalamic-pituitary-gonadal glands, and pituitary-adrenal function^42^. BPA has immunosuppressive activity^43^. PAA is probably from the crystal soil or mulches used on farms and enters animals’ bodies via animal feed. Animal feed, feed bags, storage tanks, equipment maintenance procedures, and milking equipment are the most probable origins of the MPs found in the present investigation.

Workers claim that plastic was included in the maize and animal feed straw. However, identifying a single source for MPs in livestock and poultry manure can be difficult because MPs are transferred via various pathways. Obviously, one of the sources is animal feed. However, there are indirect routes of exposure. For example, Beriot et al.(2021)^14^ reported that MPs are transported by wind to livestock. MPs can come from the soil or macroplastics ingested by animals in their stomachs and remain in manure, which may find their way into agricultural soils if composted^44^. They may also include harmful compounds adsorbed on polymers that are progressively released from their surface and enter the human food chain, in addition to altering soil characteristics and affecting living things^45^.

Several studies have identified MPs in manure of cows^46^, pigs^47^, chickens^48^, and sheep^49^. In a study by Briot et al. (2021)^14^ on microplastics in sheep manure, microplastics were found in all samples and the microplastic content in the dry sample was 997 ± 971 particles/kg, which is higher than the current study. The color variation of the detected microplastics may be due to the color variation of feed bags and plastic products commonly used in livestock and poultry farming. Many studies show that the most common types of MPs found are fibers and fragments in compost derived from manures^50–52^.

Fiber is the most abundant form in most studies: 20.8–70% of MPs in pig and cow manure, 37.5% in a mixture of animal and poultry manure, MSW, and sewage sludge^53^ and 54.1% in a mixture of sheep manure and grass^54^. Fiber was also the most abundant form in the current study.

In the current study, fragments were found in a minimum, which is in contrast to the results of Zhang et al. (2023), who found that 28.3–47.8% of MPs in pig and cow manure were fragments. Bags, pipes, and plastics lost during waste sorting and transportation can form^15^. Plastic mulch, food packaging bags, or broken plastic packaging also lead to film formation^53^. In Zhao et al.(2023) study^49^, the least number of shapes included pellets, which is consistent with the current study. Pellets can be found in daily personal care products, such as facial cleansers and toothpaste, or in product packaging^55,56^. The variation in abundance, polymer types, shape, size, and color of MPs reported in different studies reflects the diverse sources from which MPs originate. In the study by Wu. et al., 2021^57^ the average concentration of the input raw material was measured as 164.8 ± 22.3 mg/kg, which is much higher than the current study but comparable to the study by Müller et al.(2021)^58^.

In Sheriff et al.(2023)^9^, microplastics abundance (particles/kg-1) was 9.02 × 102 ± 1.29 × 103 in pig, 7.40 × 101 ± 1.29 × 102 in cow, 0 to 5000 in sheep, and 129.8 ± 82.3 in chicken manure. Microplastics that have been found were primarily ingested from feed and plastic mulching film attached to crop residues. In Wu et al.(2021)^39^ study, a total of 115 items of manure MPs and 18 items of feed MPs were identified as PP and PE types dominated by colorful fragments and fibers. Various studies showed that AMs have a high potential for fertilization in plant growth^59,60^.

The main challenge of using animal fertilizer is the increased contamination possibility of soil and water contamination with endocrine-disrupting compounds (EDCs) and AABs, which causes many environmental concerns in terms of their adverse effects^61^. Agricultural fields that employ AMs are regarded as a significant source of hormone in the environment. Agricultural fields that employ AMs are regarded as a significant source of steroid hormones in the environment^62^. Li et al.(2021)^8^ showed that the concentration of steroid hormones in COM and POM in China was 39.27 ng/kg and 40.08 ng/kg, respectively, which is lower than the current study. In Ho et al.(2014) study^63^ in Malaysia, similar to our investigation, progesterone was found in all samples, while the concentration of progesterone in animal dung in the Malaysian study was lower (8–367 ng/kg). In Bevacqua et al.(2021)^64^, the average concentration of steroid hormones in poultry manure was detected as 63 ng/kg. The differences in AABs across the study sites may be attributed to the different geographical locations of the sites, animal species, and different administration habits^65^.

In Zhang et al.(2019)^66^, 16 steroid hormones in the animal manure with concentrations ranging from 3.26 ng/g dw to 2520 ng/g dw, which is lower than the present study. Ho et ail.(2014)^63^ in Malaysia, it was shown that animal manure poses a risk of contaminating agricultural soil. Lorenzen et al.(2014) Study^67^ showed that progesterone hormone levels in animal feces were related to the type, sex, age, reproductive status of the animals, diet, and hormone treatment of the animals.

In Zhuo et al.(2020) study^68^ in China, tetracycline antibiotics were found to be the most prevalent substances in AMs, where their concentration was 1920 mg/kg, much higher than in the present research. In Hong et al.(2023) study^69^, the dominant AABs were tetracycline and EFC, which is consistent with the current study. In Alavi et al.(2015) study^70^ on the presence of antibiotic compounds in AMs in southern Iran, the concentration of tetracycline family was similar to the current study. Antibiotic levels among animal species may be in terms by variations in feed dosage levels and age, weight, and health status of animals, and so on^71^.

One of the main sources of antibiotic release into the environment is the use of livestock and poultry manure in traditional agriculture. In Iran, most of the disposed animal manure enters agricultural lands without processing, as there are no regulations requiring pretreatment of manure before application to the land. Inadequate pretreatment of waste materials and limited methods of complete antibiotic removal mean that most of the antibiotics used in livestock production industries are discharged into the environment, with an estimated 54% entering agricultural soils^72^.

In Li et al.(2021)^8^, the concentration of cadmium, lead, and nickel in AMs is usually below the WHO standard^73^, but the concentrations of Zn, Cr, and Cu were high. In Li et al.(2020)^5^ considered the sources of HMs in AMs to be livestock and poultry feed, crop contamination due to soil contamination, and feed contamination in terms of processing. Cr in COM was found to be 678.7–2.9 mg/kg, while in the current study, it was found to be 15.6–55.7. In Liu et al.(2020) study^74^, it was reported that the emission of HMs in China from animal manure would be up to 2.86 × 10^5^ tons. Zn and Cu may also enter agricultural soils through animal manure^75–77^. The detected concentrations of Zn and Cu were greater than those of other HMs for various samples of animal manure, suggesting that the issues of residual Zn and Cu in fertilizers need more study. In contrast to the present research, the average Cu and Cr levels in Liu et al.(2020) study^74^ were found to be 8.63–14.47 mg/kg and 0.05–0.78 mg/kg, respectively. The difference in average concentrations can be attributed to the diversity of livestock and poultry farming structures and dietary habits of people^78^. Feeding mode and animal farm scales had little effect on the distribution of contaminants. Using heavy metals, especially in poultry and livestock farms, should be strictly monitored to reduce potential risks to the environment and humans.

The animal manure could be accompanied by potential hazards to humans and the environment due to trace metals. In Ahh et al.(2021)^79^, chromium, copper, and zinc were analyzed, which mean concentrations were Cr 2.9 mg/kg, Cu 20.4 mg/kg, and Zn 79.8 mg/kg. The Duan et al.(2021)^19^ was in contrast with the present study in that Cu, Cr and Zn were higher than the limit of WHO standard. Also, livestock manure had high contents of Zn, Cu and Cr. Previous study^80^ has shown that the application of livestock and poultry manure increased the accumulation of Cu, Cr, and Zn in the soil.

In Xiong et al.(2010)^76^, the concentrations of Cu in pig, cattle, chicken and sheep manure in China were surveyed. The mean Cu concentrations in pig, cattle, chicken and sheep manures were 699.6, 31.8, 81.8, and 66.85 mg kg^−1,^ which were positively correlated to that in their feeds. In Austria^81^, intense additions of Cu and Zn are reflected in high loads in the manure, and these amounts in some instances exceeded the threshold limits for soil contamination.

In total, Animal manure is an important reservoir of emerging pollutants, and the use of manure on agricultural land is likely to be a major pathway for emerging pollutant contamination.

## Conclusion

For the first time in Iran, this study surveyed the emergent pollutants such as microplastics and steroid hormone in poultry and cow manure. In the current study, microplastic contamination was observed in more than 70% of the samples. Fiber shape, blue color, and polymers of PP and PE were the most common microplastics. Sphere – blue and fiber-black were maximum in POM and COM, respectively. All samples contained different amounts of AABs and PST. HMs in poultry manure were higher than in COM. The concentrations of emerging pollutants varied depending on the animal and the location of sample collection. Regular monitoring of emergent pollutants in AMs and some control measures to prevent the spread of their contamination to the environment are necessary. The limitation of this study is the lack of access to laboratory devices with nanogram detection capabilities for measuring other low-concentration pollutants. It is suggested that future studies focus on assessing the plastic in the manure of other animals, such as sheep, goats, pigs, roosters, ducks, and so on, in different regions of Iran. They should measure a wide range of heavy metals and pharmaceutical compounds in animal manure, depending on the animal, season, animal rearing period, age of animal, etc., and estimate the degradation of plastic in the intestines of livestock.

## Data Availability

The datasets used and/or analyzed during the current study are available from the first author Mitra Mohammadi, on reasonable request via e-mail mitramohammadi@kums.ac.ir.
